# Bacterial interactions underpin worsening lung function in cystic fibrosis-associated infections

**DOI:** 10.1128/mbio.01456-24

**Published:** 2024-11-22

**Authors:** Damian W. Rivett, Lauren R. Hatfield, Helen Gavillet, Michelle Hardman, Christopher van der Gast

**Affiliations:** 1 Department of Natural Sciences, Manchester Metropolitan University, Manchester, United Kingdom; 2 Department of Life Sciences, Manchester Metropolitan University, Manchester, United Kingdom; 3 Department of Applied Sciences, Northumbria University, Newcastle, United Kingdom; 4 Department of Respiratory Medicine, Northern Care Alliance NHS Foundation Trust, Salford, United Kingdom; University of Kentucky, Lexington, Kentucky, USA

**Keywords:** polymicrobial infection, cystic fibrosis, microbiome, pathogenic interactions, respiratory infection

## Abstract

Chronic lung infections are the primary cause of morbidity and early mortality in cystic fibrosis (CF) and, as such, have been the subject of a great deal of research. Subsequently, they have become one of the key paradigms for polymicrobial infections. The literature, however, has traditionally focused on the presence of pathogens in isolation or univariate measures like number of species to predict decline of lung function and ignores large swathes of data. Here, we suggest that looking at the interactions between species identified by 16S rRNA gene sequencing, rather than at species singularly, could elucidate hitherto unknown properties of these complicated infections. To confirm this, pooled samples from studies conducted by our laboratory, sequenced using the same pipeline, were used to assess microbiome-wide associations to lung function. We found pathogenic interactions between species were limited to the most abundant species, which were composed of canonical CF pathogens (including *Pseudomonas*, *Staphylococcus*, *Stenotrophomonas*, and *Achromobacter*) and commensals. This observation is crucial for better understanding of polymicrobial infections and treatment of these conditions while providing a simple framework for expanding this research into other disease states. The adoption of ecological principles into infection science can provide better understanding and options to those suffering from chronic conditions. The statistical ecology approach presented here enables clear hypotheses from observational data that can be ratified through subsequent manipulative experimental studies. Moreover, it can also be used to support the design and construction of clinically relevant *in vitro* models of polymicrobial infections.

**IMPORTANCE:**

Research studies have repeatedly demonstrated that chronic lung infection in cystic fibrosis is polymicrobial and consequently does not adhere to the single microbe-based Koch’s postulates. Despite the plethora of evidence, the role of the constituent taxa present is largely unknown. Here we demonstrate how an ecological modeling perspective on lung infection microbiota can tease out potential interactions that alter progression of disease. Using techniques akin to genome-wide association studies, we show and validate 22 taxa, present in the chronic respiratory disease associated with cystic fibrosis, which have significant interactions that are negatively associated with patient lung function, the majority of which are “non-pathogenic” organisms. This work highlights the need to understand the interactive landscapes of the microbiomes to fully appreciate the complexity and treat chronic lung infections. Furthermore, this presents testable hypotheses for manipulative experiments in model systems to elucidate key mechanisms to driving disease progression.

## OBSERVATION

Embedding ecology into the study of microbiomes is essential for understanding and predicting dynamics of the microbiota (1–3). This is highly pertinent in the case of chronic polymicrobial infections, where interactions between bacterial species alter the progression of disease. Take the systemic, genetic disorder, cystic fibrosis (CF), where infections of the respiratory tract are the leading cause of morbidity and mortality (4). Decades of research have shown there are many causative microbial agents, where microbes work in consort to progress the disease (5–8) and infectious communities are the norm (8, 9).

Previous studies indicate that there is a significant, positive relationship between the number of bacterial species present and lung function (defined as percent forced expired volume in 1 second predicted [% FEV_1_]). As the communities become more complex, the better the outcome for the patient (5, 10). Despite this significance, the reported coefficients are often weak (*R* < 0.2) (5), suggesting that the unaccounted variation within the data is due to patient variability, the presence of keystone species, and/or interactions within the microbiome. This study focuses on the interactive landscape of CF-associated respiratory infections, elucidating how interactions play a key role in the progression of disease and highlighting specific interactions that lead to worsening clinical outcomes.

The analysis used 315 samples from 112 people with cystic fibrosis (pwCF), of which 52 provided two or more samples (11, 12) (Table S1). These samples were divided into a “test” data set comprising initial samples (*n* = 112), and a “validation” data set, which contained all the remaining samples (*n* = 203). All samples were analyzed for 16S rRNA gene diversity using the same pipeline (1) and aligned to the lowest taxonomic level possible up to genus (>95% sequence similarity) level (Supplemental Methods).

**Fig 1:**
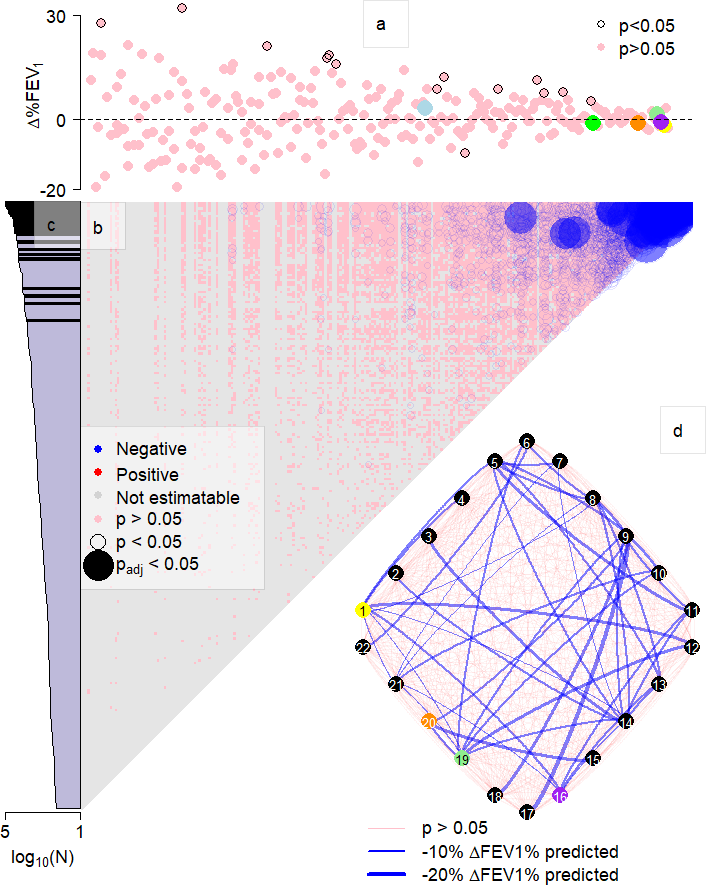
Associations between bacterial taxa and lung function. The abundance of each bacterial taxa was associated with lung function (a), indicating a range of association coefficients, which were non-significant (pink points) after Bonferroni correction. Interactions between all pairwise combinations of bacterial taxa (b) were shown to have negative associations (blue coloring) with lung function. Interactions that could not be estimated (for example, taxa did not co-occur) are the background (grey) color, with non-significant interactions also displayed (pink). Significant interactions, after Bonferroni correction, are shown by solid points with increasing size indicating greater significance and are exclusively found among taxa accounting for >0.1% of the total relative abundance (black bars, c). In total, 45 significant interactions were observed between 22 taxa. There were different numbers of associations for each taxon with differing impacts shown by increased line thickness for greater effects (d). Despite differences in magnitude, all effects were negative (blue) on lung function. The canonical pathogens are colored: *Achromobacter* (yellow), *Burkholderia* (green), *Haemophilus* (light blue), *Staphylococcus* (light green), *Stenotrophomonas* (orange), and *Pseudomonas* (purple), and taxa identified with pathogenic interactions are listed in Table S2. Given the length of the ribosomal sequences analyzed, taxonomic assignments should be considered putative.

From the 112 respiratory samples in the test data set, 259 distinct bacterial taxa with mean (±SD) of 25 (±9) taxa per pwCF were observed. The relationship between lung function (mean ± SD, 55.75% ± 22.75% FEV_1_) and microbial richness was assessed; however, no significance (*F*_1,110_ = 0.13, *P* = 0.724) was found. The data were subsequently interrogated to understand the role of individual microbial taxa on patient lung function. Here, we utilize previously championed microbiome-wide associations (MWAs) (13, 14) that have revealed previously unknown functional consortia that exist within environmental microbiomes (15). Observed associations between individual taxa abundance (sequence reads) and lung function indicated there was a broadly equal number (*n* = 136, 52.5%) of positive (i.e., the higher the number of reads associated with a taxon corresponds to a higher lung function) and negative (*n* = 123, 47.5%) associations; however, none were significant after multiple corrections were applied (Fig. 1a). This was surprising as the canonical pathogens have been found as the dominant organism in lungs of patients with end-stage disease (16); therefore, their increased abundance should be associated with worsening lung function. Our data suggested that the abundance of the canonical pathogens had no significant association with differences in lung function: *Pseudomonas* (*F*_1,110_ = 0.22, *P* = 0.636), *Burkholderia* (*F*_1,110_ = 0.17, *P* = 0.400), *Staphylococcus* (*F*_1,110_ = 0.71, *P* = 0.636), *Achromobacter* (*F*_1,110_ = 1.02, *P* = 0.314), *Haemophilus* (*F*_1,110_ = 0.53, *P* = 0.467), and *Stenotrophomonas* (*F*_1,110_=0.29, *P* = 0.590). This suggests that the abundance of a single taxon does not account for a significant amount of lung function variation in our data set. This is significant as it further questions the “one microbe, one disease” standpoint of infection pathogenesis and that the cause of lung function variation was down to other factors, for example, the mitigation of a single taxon’s presence by the plethora of co-existing taxa (15), not accounted for by the presence of these species alone.

The analysis next turned to an investigation of whether interactions between taxa impacted the lung function of pwCF. This analysis aimed to account for the presence of the rest of the community by creating statistical “pathogenic interactions.” These have been well documented between canonical pathogens in previous studies; however, these evidenced interactions are limited to low numbers of culturable taxa (17–22). While these are highly valuable and prized experiments, particularly by characterizing specific interactive mechanisms (17–22), without the inclusion of all members of the microbiota, they cannot account for missed interactions with unsuspected organisms (23). We applied another MWA search to these data, this time accounting for the presence of the all identified taxa within the community and their combined association with lung function (15). Our analysis detected 45 (0.13%) significant pairwise interactions between members, all of which associated the presence of both taxa with a reduced patient lung function (Fig. 1b). Furthermore, these significant interactions were only identified between the most abundant (≥0.1% relative abundance) taxa (Fig. 1c). To evaluate the taxa associated with these negative interactions, data were extracted for all significant interactions. These interactions were observed between 22 taxa (Fig. 1d), including 4 of the canonical pathogens: *Pseudomonas*, *Staphylococcus*, *Achromobacter*, and *Stenotrophomonas*. Experimental evidence supports our findings that combinations of pathogenic and non-pathogenic bacteria can increase the morbidity of a host (19, 22). It is still, however, prevalent to find single species linked with the progression, or even onset, of disease which we do not find evidence of in this data set using MWA methodologies.

We investigated whether these interactions were due simply to co-occurrence events. However, the significant interactions were compared, and there were significantly (*Χ*^2^_25_ = 1394.7, *P* < 0.001) fewer pathogenic interactions (*n* = 45, 0.13%) than co-occurrence associations (*n* = 391, 1.18%). Unlike the pathogenic interactions, these were normally distributed (Shapiro-Wilks normality test; *W* = 0.93, *P* = 0.110) throughout the taxa (Fig. S1) and were all positive (i.e., a higher presence of one species is associated with a higher presence of another species), suggesting that changes in bacterial abundance do not directly impact lung function for the majority of taxa.

**Fig 2:**
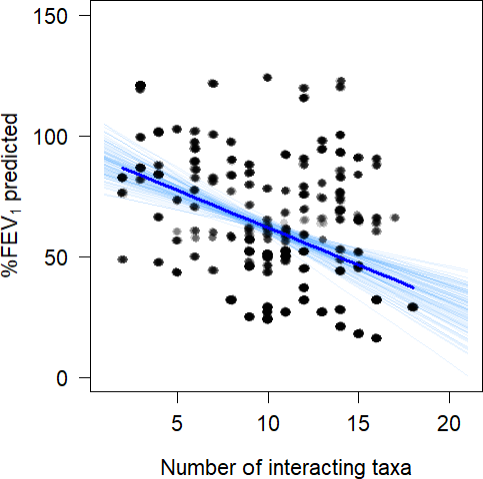
Validation of taxa with significant pathogenic associations. Random sampling (*n* = 100) of the longitudinal samples from 52 patients was undertaken, and the presence of the 22 species with pathogenic interactions was summed and plotted as the explanatory variable for lung function (FEV_1_% predicted). An increase in the number of interactive species within a sample is associated with lower lung function. Each point represents one patient sample with the corresponding lung function and the number of the interactive species present. Light blue lines represent each of the 100 associations, with the mean association displayed by the dark blue line.

At this stage, these results are inferring biological relationships from statistical models; validation of these results, in the absence of experimental models, needs to be undertaken. As such, we used longitudinal samples (1, 12) and randomly selected a single sample from each patient (*n* = 52). This random sampling was repeated 100 times, and in each instant, lung function was associated with the number of interactive taxa present. In this analysis, presence was binary and assessed regardless of abundance. The results indicated that 79% of the associations were significant (overall mean [±1 SEM] *P* = 0.035 ± 0.006) with a negative trend (mean [±1 SEM] coefficient = −3.05 ± 0.07% taxa^−1^) across the data, indicating that the more of these interactive species are present, the worse the prognosis (Fig. 2). This is an important finding as only four of these taxa are considered as canonical pathogens within CF. To further validate our results, randomly selected taxa (*n* = 22, resampled 100× without replacement) were ran through the same analysis. No significant (mean [±1 SEM] *P* = 0.279 ± 0.010) association between the number of randomly selected taxa and lung function was observed.

Previous studies have indicated that commensals, or organisms that are considered non-pathogenic and superfluous, can significantly impact organismal survival in experimental studies, but with a growing raft of microbiome data, using MWA studies, is vital for identifying potential interactions that significantly impact lung function and patient prognosis. We believe that using these data-rich microbiological surveys is key to identifying novel targets for therapeutics but first requires *in vitro* and *in vivo* experimental studies to confirm these results (24). The statistical ecology method presented here gives a clear rationale and hypotheses for *in vitro* experimentation to include the diversity of the microbiome and to move away from single-microbe investigations. Furthermore, this approach could be useful in the design and construction of clinically relevant *in vitro* models of polymicrobial infections (25).

## Data Availability

The raw data generated in this study have been deposited in the European Nucleotide Archive under the accession numbers PRJEB52183 and PRJEB62148 and in the Sequence Read Archive under BioProject numbers PRJNA1113124 and PRJNA1113142, with extra samples used in this study in PRJNA1112360. Sample information for the above data, combined with the filtered taxonomy table, is available on FigShare.com, along with the unfiltered taxonomy table and the analysis R scripts, at 10.6084/m9.figshare.25746960.

## SUPPLEMENTAL MATERIAL

Supplemental Information mbio.01456-24-s0001.docx Supplemental text, tables, and figure.

ASM does not own the copyrights to Supplemental Material that may be linked to, or accessed through, an article. The authors have granted ASM a non-exclusive, world-wide license to publish the Supplemental Material files. Please contact the corresponding author directly for reuse.
