# Supplementary material for: Bacterial interactions underpin worsening lung function in cystic fibrosis-associated infections
Source: mBio. 2024 Nov 22;16(1):e01456-24. doi: 10.1128/mbio.01456-24 (PMC11708055; doi:10.1128/mbio.01456-24)
Supplement: Supplemental Information — Supplemental text, tables, and figure. [file mbio.01456-24-s0001.docx]

**Title:** Supplementary information for “Bacterial interactions underpin worsening lung function in cystic fibrosis associated infections.”

**Authors**: Damian W. Rivett^1^*, Lauren R. Hatfield^2^, Helen Gavillet^3^, Michelle Hardman^2,4^ & Christopher van der Gast^3,5^

**Affiliations**: ^1^Department of Natural Sciences, Manchester Metropolitan University, Manchester, UK. ^2^Department of Life Sciences, Manchester Metropolitan University, Manchester, UK. ^3^Department of Applied Sciences, Northumbria University, Newcastle, UK. ^4^Current address: Microbiology and Infectious Disease, Institute of Life Science, Swansea University Medical School, Swansea, UK. ^5^Department of Respiratory Medicine, Northern Care Alliance NHS Foundation Trust, Salford, UK.

**Supplementary Methods:**

*Samples*

Samples used in this study were collected from three Cystic Fibrosis clinics located in Manchester and Southampton, UK, and Burlington, VT. All samples were collected from patients, after providing written informed consent, under ethical approval from the three centres involved. Samples from Manchester Adult CF Centre and Southampton General Hospital were collected under approval from the NHS Research Ethics Committee (Ref: 20/NW/0302 and 06/Q1704/26 respectively). Samples from Burlington, VT were collected under University of Vermont Institutional Review Board (CHRMS STUDY# M13-160). DNA extractions and 16S rRNA gene amplicons were sequenced using the same workflow as described previously^1-3^. Briefly, samples were treated with Propidium Monoazide (PMA) prior to DNA extraction by mechanical cell disruption in a Guanidium thiocyanate-EDTA-sarkosyl-phophate buffered saline lysis buffer and purification with phenol-chloroform^3^. Treatment with PMA was undertaken to remove potential bias from dead and damaged cells, and extracellular DNA which could affect the analysis^3^. From this DNA, the V5-V6 region of the 16S rRNA gene was amplified and sequenced on the Illumina MiSeq platform (300 cycle, V3 chemistry) alongside mock communities and appropriate controls^2^.

*Bioinformatics*

Sequenced samples were analysed bioinformatically using the DADA2 pipeline^4^ with filtering parameters as default except truncLen=250, maxEE=c(4,4). Taxa were assigned using the GTDB database and any sequences that were not assigned to a taxa at genus level (>95% sequence similarity) were manually inputted into BLAST^5^ (<https://blast.ncbi.nlm.nih.gov/Blast.cgi>) and assigned genera using megablast^6^. To assign putative taxonomic assignments using BLAST, only taxa that matched, or surpassed, the following thresholds: Sequence Identity > 95%, E-value < 1x10^-100^, 100% Query coverage. If more than one genus was identified that adhered to the selection criteria, then the sequence was given the original assignment from GTDB. Amplicon sequence variants assigned at Kingdom level (“Bacteria”) were removed from the analysis (n=439). All taxa were then summed together into phylotypes (the highest taxonomic assignment)^7^.

*Statistical analysis*

Initial samples were subsampled for each of the patient to form the “test” data set remaining samples were used to create the “validation” data set. Community metrics were calculated on relative abundance data using the vegan package^8^ in R (v4.3.2) along with all visualisations. Single species associations were calculated using regression models^7^ of % Forced Expired Volume in 1 second predicted (%FEV_1_) as a function of the relative abundance of a given taxon (*P_n_*), recording the coefficient *b_n_* in the equation: *y = b_0_ + b_n_*log_10_(P_n_+1)* where *y* is the %FEV_1_ measurement, *b_0_* is the intercept, and *b_n_* is the association coefficient related to taxon *n*. Co-occurrence associations were calculated using a similar regression model as described above where the relative abundance of taxon *n* (*P_n_*) as a factor of the of another taxon within the dataset (*P_x_*) in the equation: *log_10_(P_n_+1) = b_x_*log_10_(P_x_+1) + b_0_ ,* where *b_0_* is the intercept, and *b_x_* is the association between the transformed relative abundances of taxa *n* and *x*. Statistical interactions were calculated using multiple regression models for %FEV_1_ as a function of relative abundance of P_n_, P_x_ and their statistical interaction P_n_*P_x_ recording coefficient *b_i_* in the equation: *y = b_n_*log_10_(P_n_+1) + b_nx_*log_10_(P_x_+1) + b_i_*(log_10_(P_n_+1)* log_10_(P_x_+1)),* where *y* is the %FEV_1_ measurement, *b_n_* and *b_nx_* are the coefficients associated with the relative abundances of taxa *n* and *x* respectively, and *b_i_* is the coefficient associated with the interactions between taxa *n* and *x*. Coefficient *b_i_* was used as an estimate of the “pathogenic interaction” between each pair of taxa. Validation of the interactions was undertaken using regression models of %FEV_1_ as a factor of the number of interacting taxa using the equation *y = b_v_*Σ(S_1_+S_2_+…S_z_) + b_0_*, where *y* is the %FEV_1_ measurement, *b_0_* is the intercept, *b_v_* is the association coefficient for the sum of the binary presence/ absence matrix of the taxa shown to have significant pathogenic interactions (as described above) with at least one other taxon, and *S* is the binary presence (1)/ absence(0) of taxon 1 to *z* within a sample. Randomisations were performed in R with the “seed” set at 12345 and performed without replacement. All association p-values were adjusted (p_adj_) using Bonferroni corrections (p < 0.0002 for single phylotype associations; p < 1.63x10^-6^ for pathogenic interactions).

**Supplementary References:**

1. Gavillet H, Hatfield L, Jones A, et al. Ecological patterns and processes of temporal turnover within lung infection microbiota. *Microbiome* 2024;12(1):63. doi: 10.1186/s40168-024-01780-6

2. Hatfield L, Bianco B, Gavillet H, et al. Effects of postage on recovery of pathogens from cystic fibrosis sputum samples. *Journal of Cystic Fibrosis* 2023 doi: <https://doi.org/10.1016/j.jcf.2023.03.008>

3. Rogers GB, Cuthbertson L, Hoffman LR, et al. Reducing bias in bacterial community analysis of lower respiratory infections. *The ISME Journal* 2013;7(4):697-706. doi: 10.1038/ismej.2012.145

4. Callahan BJ, McMurdie PJ, Rosen MJ, et al. DADA2: High-resolution sample inference from Illumina amplicon data. *Nature Methods* 2016;13:581. doi: 10.1038/nmeth.3869

<https://www.nature.com/articles/nmeth.3869#supplementary-information>

5. Altschul SF, Gish W, Miller W, et al. Basic local alignment search tool. *J Mol Biol* 1990;215(3):403-10. doi: 10.1016/s0022-2836(05)80360-2

6. Morgulis A, Coulouris G, Raytselis Y, et al. Database indexing for production MegaBLAST searches. *Bioinformatics (Oxford, England)* 2008;24(16):1757-64. doi: 10.1093/bioinformatics/btn322 [published Online First: 20080621]

7. Rivett DW, Bell T. Abundance determines the functional role of bacterial phylotypes in complex communities. *Nature Microbiology* 2018;3(7):767-72. doi: 10.1038/s41564-018-0180-0

8. Oksanen JB, F. Guillaume ; Friendly, Michael; Kindt, Roeland; Legendre, Pierre; McGlinn, Dan; Minchin, Peter R.; O'Hara, R. B.; Simpson, Gavin L.; Solymos, Peter; Stevens, M. Henry H.; Szoecs, Eduard; Wagner, Helene Vegan: Community Ecology Package. [*http://CRANR-projectorg/package=vegan*](http://CRANR-projectorg/package=vegan) 2016

**Supplementary Table 1**: Clinical characteristics for all patients included in this study. Data are presented as mean (± standard deviation) or number and percent (%) unless otherwise stated. Where clinical information was missing, the number of patients with the data is stated after the characteristic.

| Total number of patients | 112 |
| --- | --- |
| Patients with two or more samples | 52 (46.4%) |
| Gender (male: female) | 69: 43 |
| Mean age (years) at first or only sample | 29.89 (± 11.13) |
| Age (years) range | 13 to 71 |
| Mean FEV1% predicted (%) | 54.52 (± 22.06) |
| *CFTR Genotype* | |
| Homozygous Phe508del | 54 (48.2%) |
| *Other clinical features* |  |
| Diabetes (n=103) | 42 (40.78%) |
| Pancreatic insufficiency (n=37) | 27 (72.97%) |
| *Clinic* | |
| Manchester, UK | 37 (33.03%) |
| Southampton, UK | 63 (56.25%) |
| Burlington, VT | 12 (10.71%) |

**Supplementary Table 2.** List of taxa identified with pathogenic interactions and the number of significant (Bonferroni corrected) interactions detected. Phylotypes in bold represent the pathogens, normally associated with CF airway infection.

| **#** | **Phylotype** | **Interactions** |
| --- | --- | --- |
| 1 | ***Achromobacter*** | 8 |
| 2 | *Actinomyces* | 1 |
| 3 | *Afipia* | 1 |
| 4 | *Bacillus* | 1 |
| 5 | *Bacteroides* | 7 |
| 6 | *Caenibaculum* | 3 |
| 7 | *Ezakiella* | 3 |
| 8 | *F0040* | 7 |
| 9 | *Lachnoanaerobaculum* | 9 |
| 10 | *Listeria* | 3 |
| 11 | *Microbacterium* | 4 |
| 12 | *Neisseria* | 2 |
| 13 | *Nocardioides* | 4 |
| 14 | *Porphyromonas* | 11 |
| 15 | *Proteobacteria* | 1 |
| 16 | ***Pseudomonas*** | 5 |
| 17 | *Sediminibacterium* | 1 |
| 18 | *Sphingomonas* | 1 |
| 19 | ***Staphylococcus*** | 8 |
| 20 | ***Stenotrophomonas*** | 3 |
| 21 | *Streptococcus* | 6 |
| 22 | *Veillonella* | 1 |

**
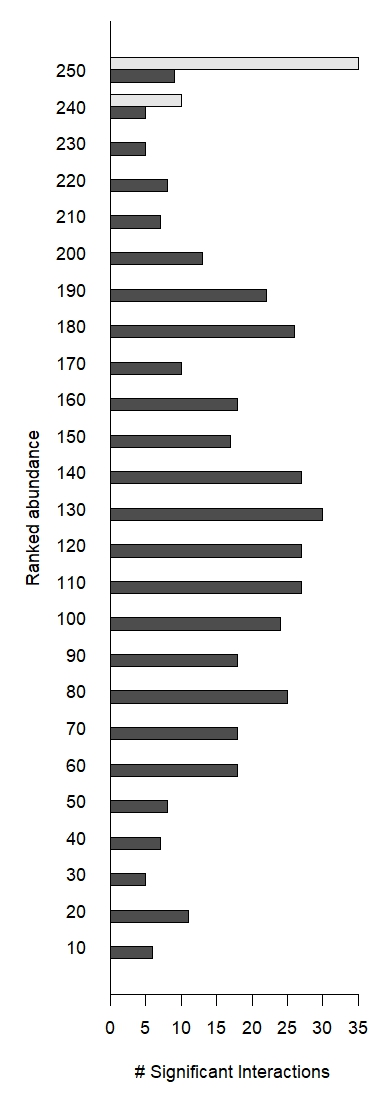
**

**Supplementary Figure 1.** Histogram showing the number of significant (p_adj_<0.05) associations between the taxa for abundance (black bars) and interactions between species impacting lung function (white bars) against the binned (n=10) ranked abundance of the taxa (1 is the least abundant, 248 is the most abundant).
